# Care across generations and borders: grandparental role and wellbeing in Sri Lankan migrant households

**DOI:** 10.3389/fpubh.2026.1778149

**Published:** 2026-07-10

**Authors:** Nipun Ranasinghe, Fahim Aslam

**Affiliations:** Marga Institute, Colombo, Sri Lanka

**Keywords:** elderly wellbeing, geriatric health, grandparental caregiving, labor migration, remittances, transnational families

## Abstract

**Introduction:**

Labor migration constitutes a critical component of Sri Lanka's economic framework, yet it necessitates complex family reorganizations in which older grandparents frequently assume primary child-rearing responsibilities. This study investigates the lived experiences of these caregivers within transnational households, evaluating the nature of their caregiving roles, the impact on their physical and mental wellbeing, and the availability of support networks.

**Methods:**

Utilizing a cross-sectional design, primary data were collected from 166 grandparents aged 60 and above from all nine provinces of Sri Lanka, using a non-probability purposive sampling approach and a standardized face-to-face questionnaire combining closed- and open-ended items. Data were analyzed using descriptive statistics, with open-ended responses examined through reflexive thematic analysis.

**Results:**

Caregivers often undertake the total substitution of parental duties, with care arrangements typically spanning 1 to 5 years. While a portion of respondents expressed emotional fulfillment and a sense of cultural duty, the study uncovers a prevalent burden of stress and physical deterioration. Nearly half of participants reported high to very high stress levels, compounded by a significant incidence of chronic non-communicable diseases, such as hypertension and diabetes, which disproportionately affect female caregivers. Over a quarter of caregivers reported receiving no assistance, relying entirely on their own resilience or limited informal networks.

**Discussion:**

Grandparental care appears to function as an important mechanism sustaining the flow of remittances and national economic stability, but it exerts a severe, often invisible toll on the older population. Recommendations emphasize the urgent need for policy interventions, including integrated geriatric healthcare, community-based psychosocial support, and targeted financial assistance to safeguard the wellbeing of these essential caregivers.

## 1 Introduction

### 1.1 Background

Employment migration has emerged as a global phenomenon that fosters economic progress, enhances human capital, and mitigates socio-economic disparities (1). In Sri Lanka, as in numerous countries in South and South-West Asia, out-migration is propelled by low per capita income, unemployment or underemployment, elevated inflation, indebtedness, and insufficient access to resources (2). Remittances significantly bolster the Sri Lankan economy, with revenues from international employment constituting the primary source of foreign currency income (3).

These changes can modify the demographic composition of homes and directly influence family methods for the care of household members simultaneously (4). Although the social ramifications of migration are reasonably understood in the context of Sri Lanka (5–7), the emphasis has been placed on children, women, and the challenges encountered by migrants throughout the migration process. According to Van Willigen and Lewis (8), Asian societies have a social expectation for grandparents to participate in the upbringing of their grandchildren. Under these circumstances, the responsibility of caring for the children left behind will inherently devolve on the grandparents. Notably, while these elderly individuals belong to transnational migrant families, much of the literature, particularly that on Global Care Chains, focuses on the cross-border dynamics between working-age migrants (often mothers) and their “left behind” small children (9). Likewise, while the positive and negative effects of grandparenting on older demographics are a burgeoning field of study (10), it remains underexplored in relation to migrant parents, where grandparents are anticipated to assume a more active and continuous caregiving role.

Owing to increased life expectancy, the percentage of older adults, including grandparents, has escalated globally; it is estimated that around 13% (1 billion) of the world population today comprises grandparents (11). In Sri Lanka, fewer than one million older adults were recorded in 1981. Nonetheless, their population increased to 1.7 million by 2001. The older population of 2.5 million recorded in 2012 is projected to rise to 5.2 million by 2037, effectively doubling during a span of 25 years (12). This population-level transformation, referred to as a demographic shift or revolution, signifies advancement in various socioeconomic variables. It signifies reductions in mortality and enhancements in longevity attributable to the advancement of Sri Lanka's public health system (e.g., the eradication of malaria), alongside intrinsically gendered transformations such as improvements in family planning (e.g., increased access to contraception), a decrease in fertility rates, and the escalating participation of women in the workforce (13).

Sri Lanka's employment migration is both prevalent and on the rise, while the country simultaneously undergoes a swift aging process driven by the population transformation described above. This dual dynamic provides a distinctive framework to examine the function of grandparents to examine the function of grandparents as primary and secondary caregivers in migrant families and its impact on their wellbeing. This study examines the dynamics of caretaking arrangements in migrant homes and their effects on the wellbeing of the elderly, aiming to comprehend the lived experiences of grandparents as caretakers from their own viewpoint.

### 1.2 Labor migration trends In Sri Lanka

Labor migration has emerged as an important aspect of Sri Lanka's socio-economic framework, undergoing considerable transformation since the nation liberalized its economy in the late 1970s. In the late 1970s and early 1980s, migration was mostly characterized by male construction and skilled laborers reacting to the Gulf region's oil boom (14). By the late 1980s, a notable “feminization” of the migrant labor force transpired, with women, pre-dominantly as domestic workers, representing about two-thirds of all departures (15). This transition aligned with an increasing demand for local labor in host nations.

The dependence on low-skilled and semi-skilled immigrant labor, especially in the Middle East and Southeast Asia, has persisted as a trend (16). For decades, the pre-dominant demographic of Sri Lankan migrants has been engaged in low-skilled professions, with female domestic workers, frequently referred to as “housemaids,” constituting a substantial segment. Data from the Sri Lanka Bureau of Foreign Employment (SLBFE) indicates that in 2023, low-skilled workers constituted a significant segment of the overall registrations (17). The pre-dominance of female workers in low-skilled categories has elicited worries about protection, inadequate remuneration, and susceptibility (18).

**Figure 1:**
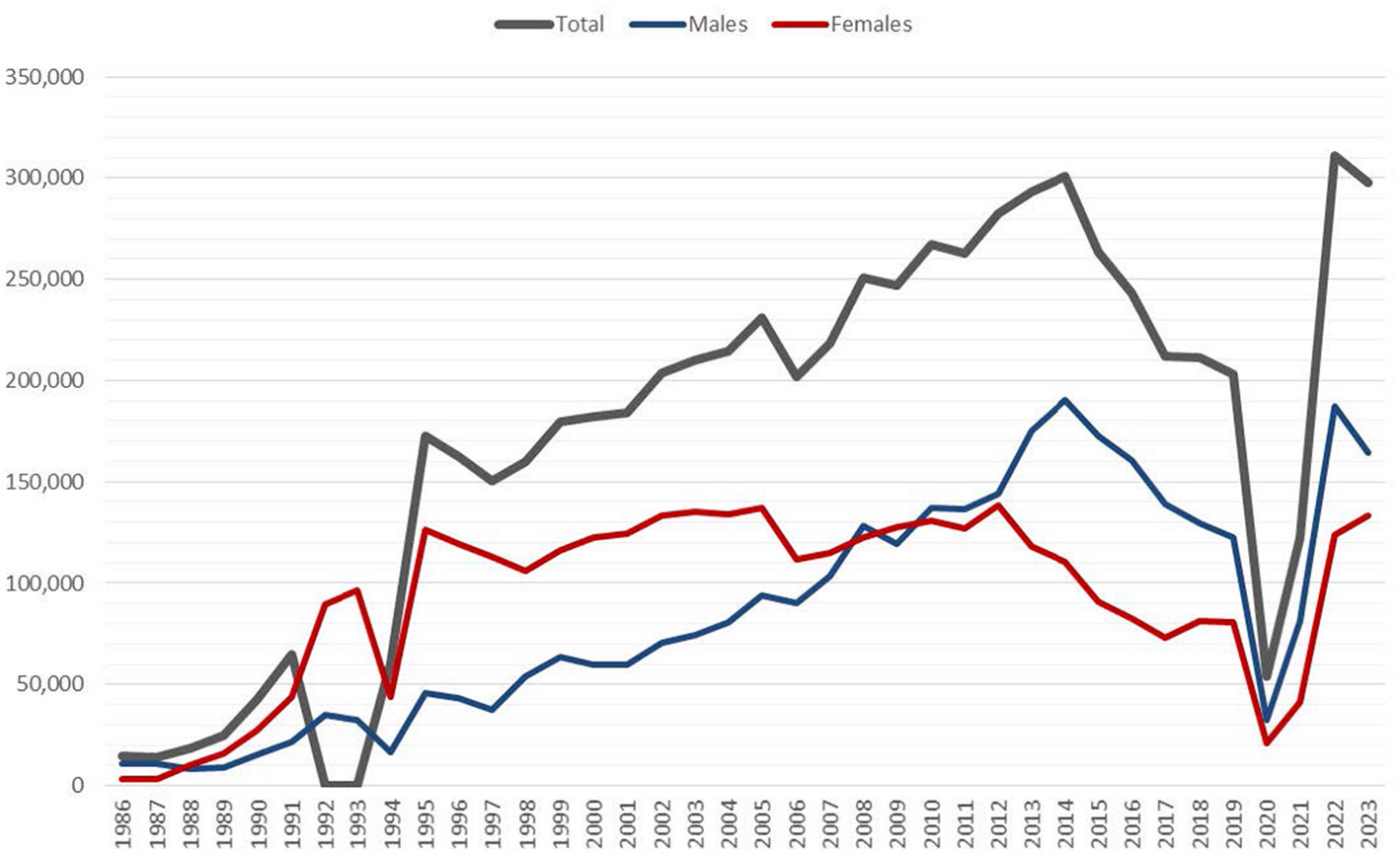
Sri Lanka's foreign employment by gender. Source: developed by the author based on the annual statistics of foreign employment 2023, Sri Lanka bureau of foreign employment.

Recently, there has been a significant policy shift focused on encouraging the migration of professional male workers while decreasing the emigration of low-skilled female workers. Numerous governmental measures, including the elevation of the minimum age for domestic employment and the implementation of compulsory occupational credentials, have resulted in a notable decrease in female labor migration (19). The tendency of female-dominated migration, previously dominant for many years, has reversed (Figure 1). In 2023, male registrations for overseas employment constituted 55.30%, whereas female registrations accounted for 44.70% (17). This policy-driven transformation is evidenced by the rising number of male migrants seeking skilled and semi-skilled employment, especially in nations such as South Korea, Malaysia, and Qatar (20).

The Middle East continues to be the principal destination for most Sri Lankan migrant laborers. In 2023, Kuwait, Qatar, Saudi Arabia, and the United Arab Emirates emerged as the primary destinations for Sri Lankan migrant labor (17). Despite a heavy dependence on this region, there is an escalating diversification of destinations, with East Asian nations such as South Korea and Japan becoming progressively appealing for skilled and semi-skilled male migrants (19, 58).

### 1.3 Implications of labor migration

Migrant workers' economic contributions, chiefly via remittances, represent the principal source of foreign money for the nation, playing a crucial role in macroeconomic stability and household sustenance (19). Research has established a substantial bidirectional causation between remittances and economic growth in Sri Lanka, indicating the profound integration of migration within the national economic structure (21). The most immediate and most examined effect is economic, chiefly via the mechanism of remittances.

Studies indicate that these cash inflows substantially aid in alleviating poverty and enhancing the material welfare of households left behind (22). Research conducted in several regions, including Pakistan and Latin America, has demonstrated that remittances alleviate the intensity and severity of poverty (23, 24). These monies are frequently employed to enhance housing, acquire land, and facilitate daily consumption, serving as a crucial economic lifeline (25, 59). In addition to consumption, remittances may be allocated to productive investments and human capital, with research indicating that this income is pre-dominantly utilized for education and health, hence improving the life prospects of the subsequent generation (22, 26).

The migration of labor across international borders can provide conflicting consequences for migrants, their families, and their communities of origin. The transformative potential of remittances is frequently hindered by existing social and structural constraints. Although individual households may attain enhanced material conditions, this does not necessarily result in overarching community-level advancement. The inability of remittances to finance enhancements in public infrastructure, such as educational institutions or sanitation, is prevalent, as migration-induced transformations frequently occur at the micro-level and fail to address structural deficiencies without deliberate state investment (27). In areas marked by limited economic prospects and patriarchal gender dynamics, the advantages of migration are frequently distributed according to pre-existing inequalities (25).

The social and psychosocial ramifications of migration, especially for family dynamics and welfare, are significant. The absence of a family member, particularly a father, results in “transnational families,” which incur significant “emotional costs” (5, 28). The extended absence of a parent constitutes a substantial stressor that may result in familial disintegration, marital discord, and mental turmoil for those remaining (6, 59). Although male out-migration may enhance the decision-making responsibilities of left-behind women, this phenomenon is not universally liberating; many women perceive this transition as a transient and onerous obligation rather than a source of empowerment (29). The elevated economic status of male migrants can disrupt conventional marital customs, enabling them to utilize their newfound status to exert greater agency in marriage and divorce, thereby undermining women's autonomy and heightening their vulnerability (25).

Gender is a significant element of this migratory movement. The process has been marked by a significant “feminization,” with a considerable influx of women relocating for domestic employment in the Middle East (30). This has sparked a challenging discourse on women's empowerment and welfare. Handapangoda (30) further elucidates that, although migration affords access to independent income, this economic agency is frequently controlled and restricted by patriarchal systems within the family and society. The absence of mothers may incur high societal costs and provoke a “masculinity crisis” among husbands who become financially reliant, perhaps resulting in the misappropriation of remittances, alcoholism, and marital instability (31). In this environment, the extended family's role, especially that of female relatives, is crucial for childcare and household maintenance, highlighting the robustness of traditional kinship structures in supporting migration in Asian nations such as Sri Lanka (32).

The impacts on children are especially pronounced and thoroughly established. Parental absence is regularly associated with adverse psychological effects, such as diminished mood, anxiety, and depressed symptoms in left-behind spouses, as well as behavioral issues in children (59). Although remittances can enhance educational prospects, this advantage is frequently counterbalanced by the detrimental impacts of lacking parental attention, affection, and direction (5, 6). Research in Sri Lanka has shown several detrimental effects on children, such as emotional distress, subpar academic achievement, and heightened susceptibility to maltreatment (7). This engenders a paradox wherein migration pursued to ensure a more favorable future for children may concurrently hinder their immediate emotional and social development, presenting a significant dilemma for “labor sending” nations like Sri Lanka (59).

### 1.4 The role of grandparents as caretakers

The demographic composition of contemporary families is increasingly influenced by labor migration, resulting in a notable prevalence of “skipped-generation and transnational households.” These families, in which grandparents take on the primary or secondary caregiving responsibilities for their grandchildren due to the absence of parents, constitute a significant, albeit frequently challenged, family structure (33). Parental migration, influenced by socio-economic “push” variables such as domestic poverty and “pull” considerations such as the potential for increased earnings abroad, is the principal driver of family formation (3).

The choice for grandparents to assume major or secondary caregiving roles is complex and frequently influenced by cultural conventions, economic imperatives, and the absence of feasible alternatives. Researchers have established a valuable typology to elucidate these motivations, classifying grandparents as “child savers,” who rescue children from familial crises; “mother savers,” who provide childcare to facilitate mothers' workforce participation; and “family savers,” who offer supplementary support (34). Within the realm of labor migration, especially in numerous Asian societies characterized by robust familial connections, the notion of the “family maximizer” holds significant relevance. In this context, the provision of childcare by grandparents is an essential component of a familial strategy aimed at augmenting economic resources, enabling their adult offspring to relocate for employment with the anticipation of receiving financial support through remittances (35). This caring function arises from cultural norms of filial piety and intergenerational duty (36). Nevertheless, the reality frequently resembles an easily accessible arrangement rather than a genuine decision. The lack of inexpensive and reliable formal childcare alternatives, especially in rural regions where many migrants come from, results in grandparents being the sole accessible caretakers (37). The acceptance of this duty involves inherent complications. The procedure may be begun by the grandparents, requested by the migrating adult offspring, or, in more painful situations, arise from the open desertion of children, necessitating the grandparents to assume responsibility (36). As a result, grandparents are compelled to assume responsibilities that surpass conventional grandparenting, encompassing financial administration, educational oversight, and comprehensive parental duties.

The structural and relational dynamics of this caregiving are further characterized by a pronounced matrilateral bias, a tendency that is magnified by the social realities of transnational migration. When adult children migrate for international labor, the responsibility for “left-behind” children in skipped-generation households overwhelmingly falls to maternal grandmothers (37, 38). Sociologically, this asymmetry is driven by strong intergenerational mother-daughter solidarity and the demand for highly trusted care arrangements that allow migrant women to confidently sustain their roles as distant providers (5, 28). This practical reliance aligns with theories of preferential investment, which demonstrate that maternal grandmothers possessing absolute relationship certainty consistently provide the highest levels of childcare (39, 40). By absorbing the daily demands of raising grandchildren, these grandmothers perform a vital “load-lightening” function that directly enables their migrant daughters' economic productivity, yielding benefits that accrue exclusively to the maternal line (41). As a result, maternal grandmothers emerge as the indispensable linchpins of global care chains, operating robustly even “under the radar” within societies traditionally governed by strict patrilineal or virilocal norms (42). Ultimately, however, this intersection of cultural expectation and social tendency dictates that the exhausting physical and emotional tolls of international migration's care deficits are disproportionately shouldered by aging women on the maternal side of the family (43).

### 1.5 Implications of caretaking

Grandparents in migrant households encounter numerous problems that profoundly affect their physical, emotional, and social wellbeing while supporting their families. The presumption of custodial care in these conditions is an acknowledged risk factor for psychological distress (44). Financial anxiety is a prevalent and significant aspect of their existence. This strain is dual in nature. The apprehension over the economic stability of their migrant children and the anxiety about the sufficiency of remittances to meet the household's requirements (45). Research on the daily experiences of caregiving grandmothers indicates that financial issues, including expenses for food, household bills, and medical necessities, consistently generate stress (46). The expenses related to raising grandkids, encompassing schooling and healthcare, are considerable and frequently surpass the restricted income of the grandparents and the remittances they obtain (47).

This financial strain is exacerbated by considerable physical and mental health issues. The obligations of full-time caregiving substantially impact the health of elderly individuals. Studies consistently indicate that grandparent caregivers experience elevated levels of sadness and inferior physical health relative to their non-caregiving counterparts (48, 49). The physical requirements of caring for young children can be arduous and fatiguing, leading many grandmothers to concern themselves with their ability to sustain the caregiving tempo as they age and experience health deterioration (50). This physical strain is exacerbated by psychological and emotional difficulties. The notion of “worry” as an automatic and negatively valenced cognitive activity is fundamental to their experience (60). This persistent anxiety has been demonstrated to adversely affect both physical and mental health (51). Grandparents express anxiety, poor mood, and depression symptoms directly associated with the everyday pressures of their responsibilities and worries for the welfare of their migrating children and the grandchildren they care for (3).

The reorganization of the family may also engender considerable relational tensions. A primary source of stress is the connection with the estranged adult child. Grandparents frequently express concern regarding mental and physical alienation from their offspring (45). Moreover, migration may result in the disintegration of traditional familial structures and societal conventions, engendering tension and conflict (31). Intergenerational conflict between grandparents and adult parents, particularly regarding child-rearing techniques during visits, constitutes a significant stressor associated with an elevated risk of depression for both groups (52). The situation is exacerbated by social isolation, since caring responsibilities can restrict grandparents' engagement in community events and hinder the maintenance of social networks, resulting in significant loneliness.

Notwithstanding the substantial evidence of adversity, the experience of grandparent caring is not consistently detrimental. For several older parents, rearing their grandkids offers a revitalized sense of purpose and camaraderie, mitigating feelings of loneliness that are prevalent in later life (36). In numerous Asian and African cultures, where familial care is highly esteemed, grandparents frequently do not view their tasks as burdensome and instead find fulfillment in performing their familial obligations (43). The emotional bond with their grandkids can be a substantial source of joy. Nonetheless, the evidence clearly indicates that these happy emotions coexist with substantial financial, physical, and emotional difficulties.

The current research on migration in Sri Lanka is deficient in studies examining the perspectives of elderly caretakers inside migrant households. The proposed study aims to investigate the nature of their responsibilities and the impact of these positions on their lives, encompassing both negative and positive effects. The study is anticipated to possess significant relevance, given Sri Lankan society is undergoing swift population aging and out-migration, which have altered the prevailing household dynamics.

### 1.6 Some methodological literature

Research on the role and welfare of grandparents in migrant and transnational households utilizes many methodological approaches, including extensive cross-national surveys and detailed ethnographic studies. Quantitative designs are often employed at a macro level to identify structural patterns in living arrangements and resource distribution. Das and Zimmer (33) utilize worldwide comparative research to ascertain the relationship between skip-generation homes, frequently arising from the migration of adult offspring, and wealth and economic security. Baker and Silverstein (35) employ survey data to examine caregiver wellbeing across several cultural contexts, including China and the United States, enabling researchers to discern the impact of national context on the relationship between caring roles and health outcomes. In Southeast Asia, Knodel and Nguyen (43) and Ingersoll-Dayton et al. (36) enhance survey-based methodologies to delineate specific “pathways” of support, quantifying the material and instrumental exchanges between migrant children and the grandparents who remain to care for the grandchildren.

In addition to these extensive statistical methods, researchers utilize focused comparative and correlational designs to assess individual psychological outcomes. Strawbridge et al. (49) illustrate the efficacy of comparative group techniques by juxtaposing grandparents with spouse and adult-child caregivers to discern distinct role-specific stresses. Kelley et al. (47) employ correlational designs to experimentally examine the relationships among physical health, social support resources, and psychological discomfort among grandmother caregivers. The literature pre-dominantly uses qualitative and anthropological methodologies to explore the lived experience of transnational separation that surveys frequently overlook. Gamburd's (38) ethnographic research in Sri Lanka offers a vital methodological instrument for examining “kin relations,” demonstrating how the dynamics of international labor migration (53) contemporaneously alter family responsibilities. Furthermore, researchers have transcended conventional retrospective interviews by implementing novel data collection methodologies; Musil and Standing (46) utilize daily diaries to document the variances in caregiving experiences, while Caldwell et al. (52) adopt multi-informant designs to examine family dynamics from the dual viewpoints of both the grandmother and the younger generation.

## 2 Methods and materials

### 2.1 Study design

This study is based on primary data collected through an independent cross-sectional survey conducted across all nine provinces of Sri Lanka. Primary data collection was necessary given the absence of any existing national survey that simultaneously captures labor migration status and grandparental caregiving arrangements among older adults.

### 2.2 Study population

The target population comprised individuals aged 60 years and older who served as primary or secondary caregivers for children under 18 in households where at least one parent was employed overseas. Eligibility was not restricted by gender, marital status, or place of residence, provided respondents met the age, caregiving, and migration-related household criteria.

### 2.3 Sampling procedure

Given the absence of a comprehensive sampling frame for this population, a non-probability purposive sampling approach was employed. Respondents were identified through field-based outreach, with Grama Niladhari officers and community contacts facilitating access to eligible households. SLBFE divisional records assisted recruitment in some urban areas. Respondents were recruited directly in the field across urban and rural settings in all nine provinces, with deliberate variation ensured across geographic location, income levels, and household size. While this approach does not yield a statistically representative sample of the broader elderly population, it is appropriate for research targeting a structurally defined subgroup not systematically enumerated in national registers. A total of 166 valid responses were obtained after excluding incomplete questionnaires. The margin of error was estimated at ±7.61% using the standard formula for a large population at a 95% confidence level (Raosoft sample size calculator), reported here as a benchmark of sample adequacy rather than as a property of any specific estimate.

### 2.4 Data collection instrument

Data were gathered via a standardized questionnaire, administered via face-to-face interviews conducted by trained data collectors. The questionnaire comprised both closed-ended and open-ended questions to obtain quantitative data and contextual insights. The instrument encompassed essential domains such as household and caregiver demographics, migration patterns of adult children, the extent and nature of caregiving duties, challenges faced in caregiving, sources and types of support from familial and social networks, and caregivers' self-reported physical and mental health status.

Before data collection, data collectors received training to guarantee uniformity in questionnaire delivery and ethical interaction with older participants. Participation was optional, and informed consent was acquired from all participants. Interviews were conducted with consideration for the participants' age and health status.

### 2.5 Data analysis

The analysis primarily employed descriptive statistical techniques to summarize demographic characteristics, caregiving roles, health conditions, stress levels, and support structures among caregiving grandparents. Frequencies, percentages, and summary distributions were used to present key findings clearly. Appropriate visualization techniques, including bar charts and tables, were used to illustrate key patterns and facilitate the interpretation of results. Data analysis was conducted using standard statistical software.Open-ended responses were analyzed inductively following reflexive thematic analysis framework. Initial codes were generated from the response corpus, grouped into themes, and reviewed by a second researcher to reduce interpretive bias.

### 2.6 Ethical considerations

Given the involvement of older participants, particular attention was paid to ethical considerations. Respondents were informed about the purpose of the study, assured of confidentiality, and informed of their right to withdraw at any point. No personally identifiable information was included in the analysis or reporting.

### 2.7 Limitations

Several limitations of the study should be acknowledged. First, the use of non-probability quota sampling limits the generalizability of findings to the broader population of older caregivers in Sri Lanka. Second, the study relies on self-reported measures of physical and mental health, which may be subject to reporting bias or individual perception. Third, the absence of a control group of elderly caregivers in non-migrant households restricts the ability to directly attribute observed outcomes solely to migration-related caregiving arrangements. Finally, the cross-sectional nature of the study does not allow for causal inference or assessment of changes in caregiving experiences over time.

## 3 Findings

### 3.1 Demographic and economic characteristics of migrant households

The demographic data for left-behind households reveal an average household size (excluding migrants) of roughly four members (*M* = 3.93, *SD* = 1.05) (Table 1). Concerning the structure of these households, more than half (51.50%) indicate that just one parent is presently employed overseas. The remaining portion is attributed to more intricate caring frameworks: 34.73% are designated as Single-Parent-Migrant Families, in which the children's sole parent resides abroad. 13.77% of these households are experiencing a situation in which both parents are overseas, so imposing the entire responsibility of child-rearing on extended family members or non-parent guardians. The financial stability of these households is pre-dominantly reliant on earnings from overseas migration. Foreign remittances constitute the principal income source for a substantial majority, sustaining 70.06% of households. Local economic activities function as supplementary revenue sources, with formal employment or salary constituting the subsequent most prevalent source at 18.40%. Self-employment or enterprise constitutes 12.27% of income sources. Agriculture/farming and local wage labor each account for 9.82%, but government or social welfare programs are the least utilized source, reported by 6.13% of households.

**Table 1:** Characteristics of the sample.

Location status	Total count	Female	Male	20,000–40,000	40,001–60,000	60,001–80,000	80,001–120,000	120,001–200,000	Above 200,000
Rural	72	51	21	12	11	13	8	15	13
Semi-Urban	52	39	13	6	8	6	12	6	14
Urban	42	33	9	7	7	9	3	7	9
Grand total	166	123	43	25	26	28	23	28	36

Levels of income (LKR).

### 3.2 Migration characteristics of adult children

**Figure 2:**
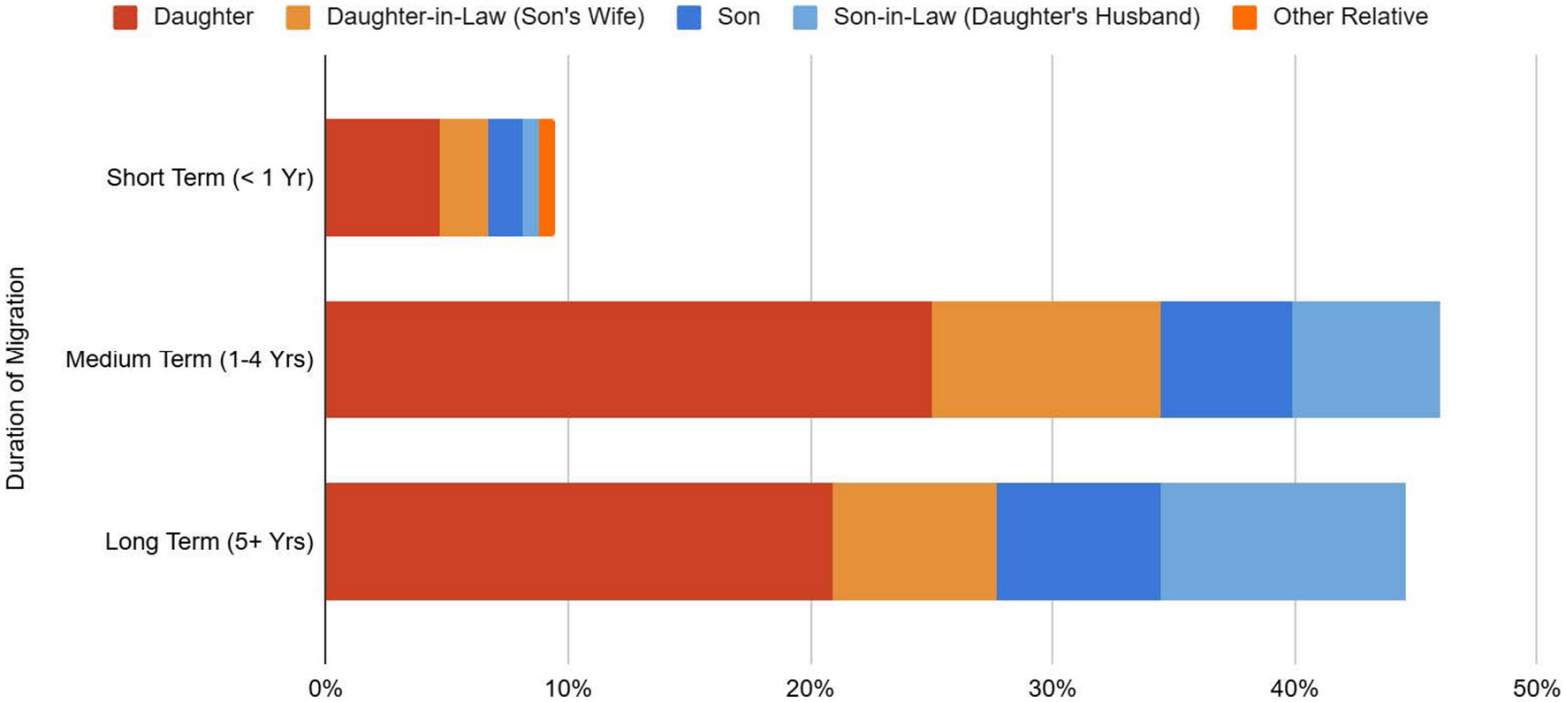
Duration of migration by relationship to caregiver (*N* = 163). Source-sample survey, 2025.

The findings on the duration of migration indicate that the financial support for families is predicated on sustained, long-term parental absence. A majority of the migrant parents were found to be abroad for medium (1–4 years) or long (5+ years) terms (Figure 2). Daughters constituted the largest single group of migrants, with 25% being abroad for a medium term and 21% for a long term, indicating a pre-dominance of maternal grandparental caregiving arrangements consistent with the matrilateral bias documented in the broader literature. The data shows a significant commitment to long-term migration across all key relationships, with 10% of Sons-in-Law and 7% of both Sons and Daughters-in-Law having been abroad for 5 years or more. Short-term migration of less than 1 year was found to be minimal across all relationship categories, which suggests that Grandparents become full-time caretakers in the event of the medium to long-term absence of parents. A precise breakdown of maternal vs. paternal grandparent status was not captured as a discrete variable in the survey instrument, which is acknowledged as a limitation of the current study.

**Figure 3:**
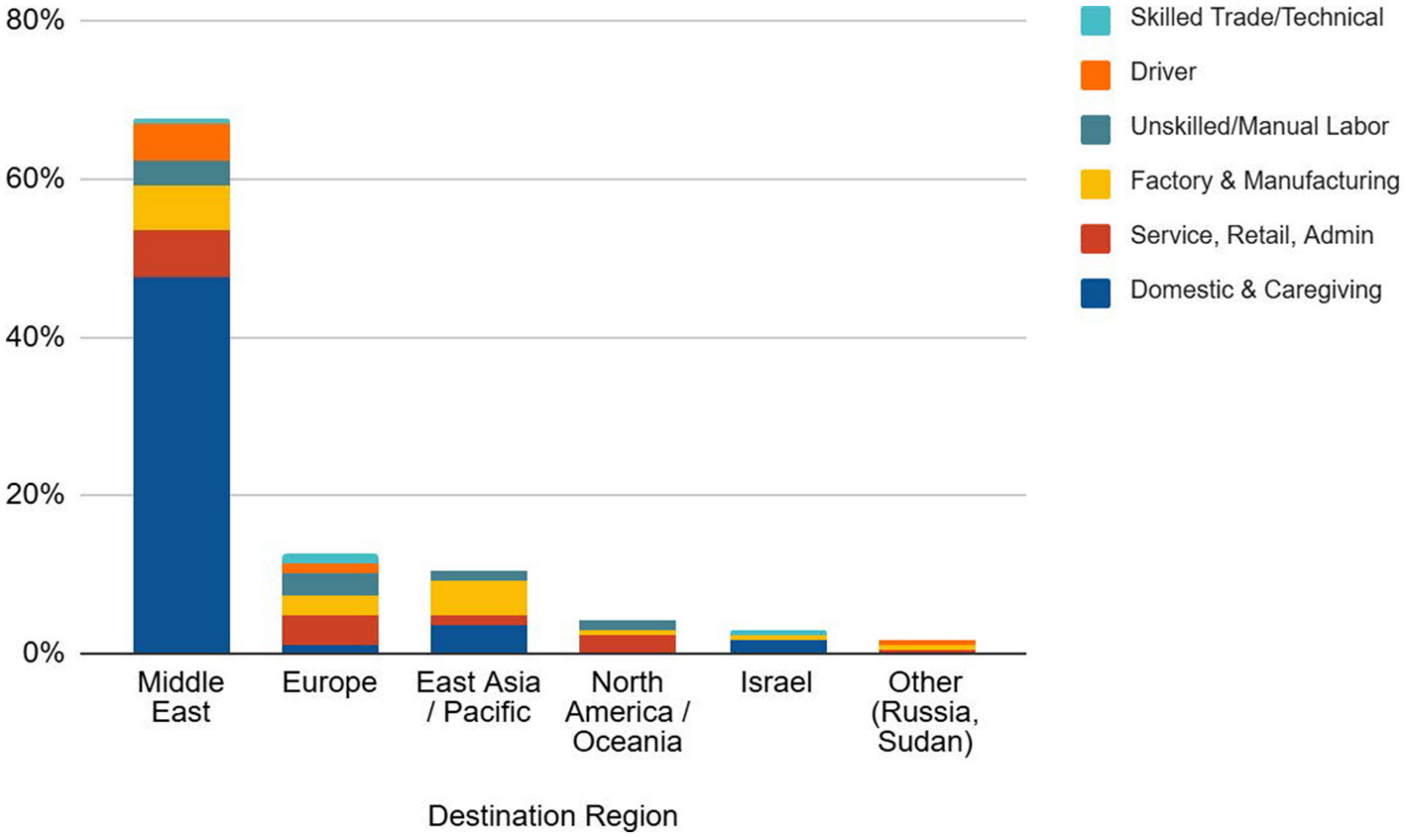
Migrant destinations by occupation (*N* = 163). Source: sample survey, 2025. The asterisk denotes that the data presented is categorized according to occupation and destination region, as detailed in the note below. Six occupational categories are utilized: domestic and caregiving, covering roles like housemaid, child caregiver, and elder care; service, retail and admin, which includes customer-facing, retail, hospitality (chef, waiter), and professional white-collar jobs (manager, accountant); factory and manufacturing, encompassing industrial and production line work such as garment worker and machine operator; unskilled/manual labor, which captures general labor, cleaning, construction, and agricultural work; driver, covering all professional driving roles; and skilled trade/technical, which requires specialized certification for trades like plumbing, electrical work, and technical mechanics. These occupations are mapped across defined geographical regions, where the Middle East includes Kuwait, UAE (Dubai), Saudi Arabia, Qatar, Jordan, Oman, and Bahrain; Europe encompasses Germany, Italy, Cyprus, Denmark, Albania, and Romania; East Asia / Pacific includes South Korea, China, and Japan; North America / Oceania covers Canada and Australia; Israel is maintained as a separate analytical category due to its unique labor market structure; and a final category groups other smaller markets such as Russia, Sudan, Singapore, and Malaysia.

The employment of the migrant parents is remarkably concentrated, both occupationally and geographically (Figure 3). The Middle East is the primary destination, and the Domestic and Caregiving sector was the dominant form of employment. Forty eight percent of *all* reported migrant occupations across *all* destinations fell into the category of Domestic and Caregiving roles, specifically within the Middle East. This indicates a primary dependence on feminized, service-sector labor in a single geographic region. In comparison, other regions were found to be secondary employment sources with different occupational specializations. Europe was a source of employment in Service, Retail, and Administration (4% of total migrants), while East Asia and the Pacific provided opportunities in Factory and Manufacturing (4%). Across all destinations, there is a near-total absence of high-skilled labor. Skilled Trade and Technical positions are statistically negligible, rarely accounting for more than 1% of occupations in any given region. The findings confirm that the employment profile of the migrant parents is mostly concentrated in low-to-mid-skilled labor sectors.

### 3.3 Health status of elderly caregivers

**Figure 4:**
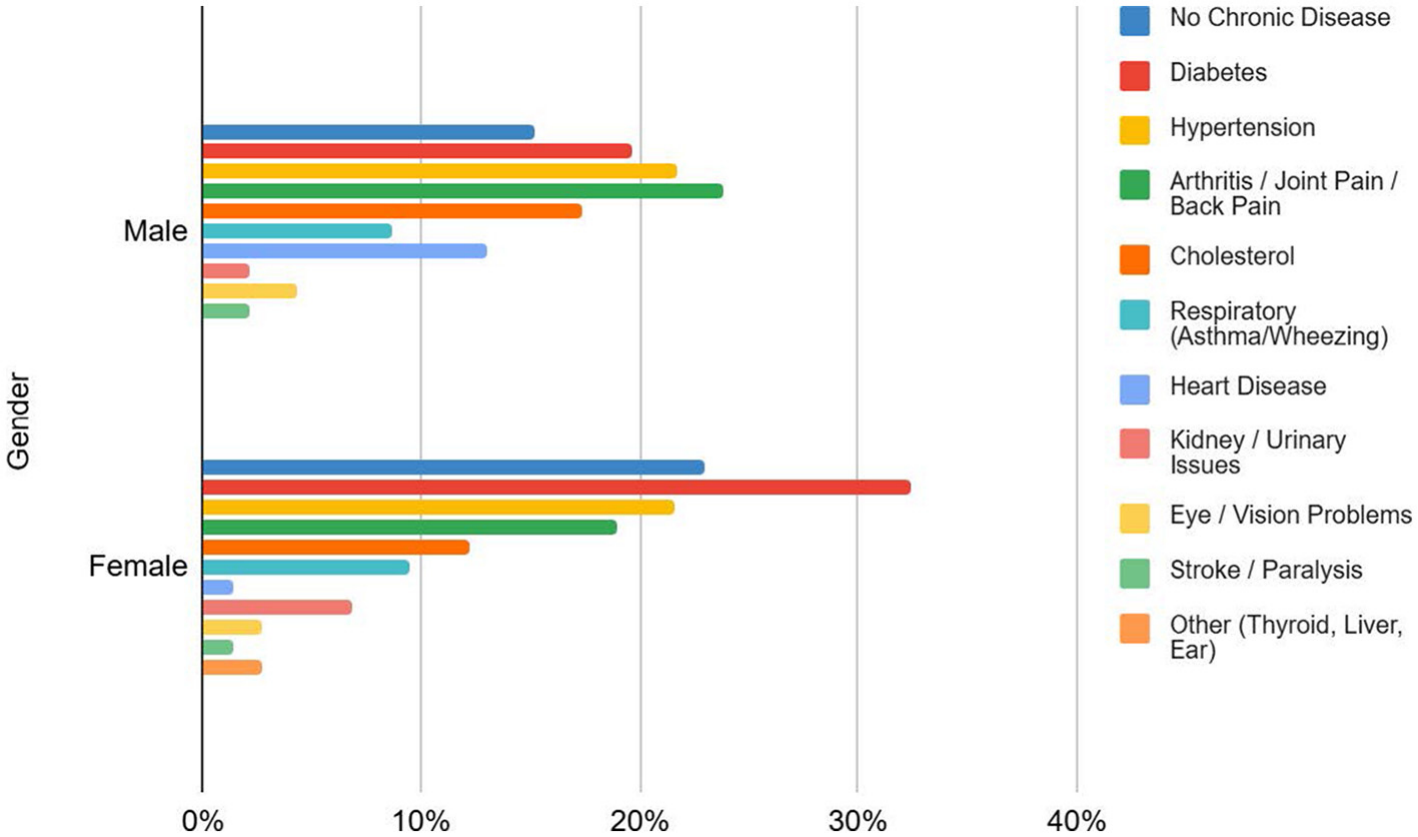
Self-reported chronic health conditions of older caregivers by gender (*N* = 163). Source-sample survey, 2025.

Self-reported health studies of elderly caregivers reveal a substantial and pervasive burden of chronic non-communicable diseases (Figure 4). The most often reported disorders are Hypertension, impacting around 21.7% of males and 21.6% of females, and Arthritis/Joint Pain/Back Pain, which affects 23.9% of males and 18.9% of females. Diabetes is a significant health concern, with an overall average prevalence above 26%. Moreover, cholesterol-related problems impact 17.4% of males and 12.2% of females, while heart disease continues to be a significant concern, particularly among men. These data indicate that the management of pain, movement restrictions, and major metabolic and circulatory disorders mostly influences the everyday health experience of caregivers.

The results indicate gender-specific patterns of morbidity, highlighting distinct health vulnerabilities for male and female caretakers. Senior female caregivers in this sample report a notably high incidence of metabolic diseases, with diabetes affecting 32.4% of female respondents, and kidney or urinary disorders three times more prevalent among women than men. The self-reported prevalence of diabetes among females is 32.40%, significantly above the 19.60% observed in males. Kidney and urinary disorders are three times more prevalent in females (6.80%) than in males (2.20%). In contrast, male caregivers endure a heightened prevalence of cardiovascular and musculoskeletal disorders. The self-reported prevalence of heart disease in males is 13.00%, about 10-fold the 1.40% prevalence in females.

Males exhibit a greater frequency of arthritis and joint pain at 23.90%. Notwithstanding these disparities, Hypertension exhibits about an equivalent frequency among all genders.

**Figure 5:**
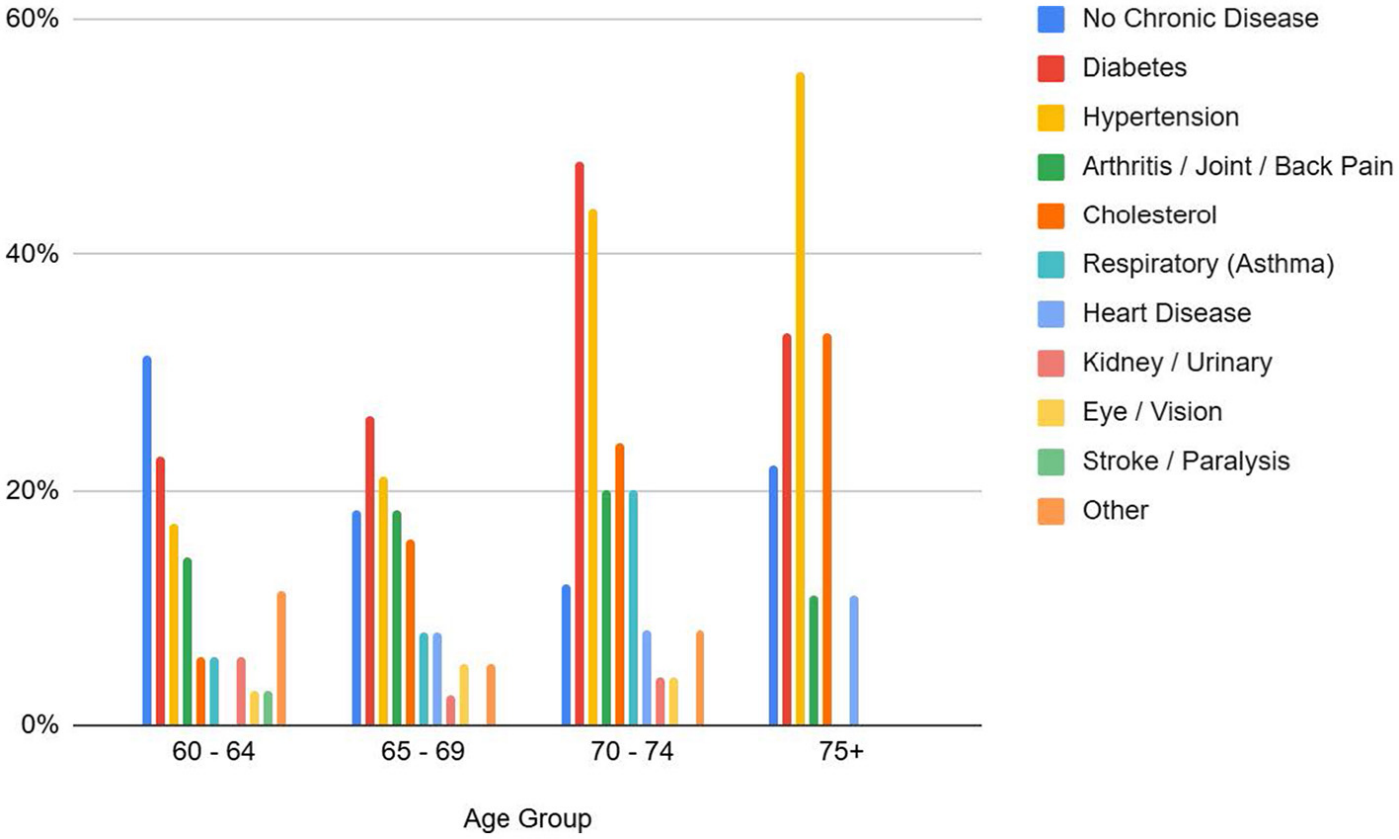
Self-reported chronic health conditions of older caregivers by age groups (*N* = 163). Source-sample survey, 2025.

The data indicate a distinct decline in self-reported health status with increasing age (Figure 5). The proportion of caregivers without chronic disease declines markedly, from 31% in the 60–64 age range to merely 12% in the 70–74 age group, indicating that chronic illness becomes prevalent among the majority of caretakers post-70 years of age. Hypertension escalates with age, reaching a prevalence of 56% in individuals aged 75 and above. The prevalence of diabetes is highest in the 70–74 age group, impacting a significant 48% of adults within that demographic. The results indicate a significant prevalence of comorbidity among older caregivers; the 70–74 age demographic exhibits concomitant peaks in Diabetes, Hypertension, high Cholesterol (24%), and Respiratory conditions (20%). The prevalence of numerous chronic illnesses among the oldest age groups indicates that the most senior caregivers are also the most medically susceptible.

For context, NCDs are already highly prevalent among Sri Lanka's general older population. Without a non-caregiving control group, no causal inference can be drawn. Chronic illness should be understood as a pre-existing vulnerability that compounds caregiving burden.

### 3.4 Nature of grandparental caregiving

The themes were discerned using a thematic analysis of the comprehensive daily activity descriptions elicited from an open-ended inquiry (What is the nature of your work caring for your grandchildren?). This entailed reviewing diverse levels of information (ranging from concise lists to extensive narratives) and categorizing replies according to the fundamental purpose or nature of the indicated responsibilities. The identified themes reveal distinct patterns: some grandparents undertake Total Substitution of parental roles (Theme 1), others emphasize Academic Oversight and logistics (Theme 2), many integrate intensive caregiving with Domestic/Economic Work (Theme 3), some collaborate with a local parent (Collaborative/Defined Support-Theme 4), and a few unique cases underscore care for Special Needs or minimal responsibilities (Theme 5) (Table 2).

**Table 2:** Nature of caretaking and its frequency (*N* = 163).

Theme	Percentage
1. Total substitution of primary parental duties	32.14%
2. Logistical management and academic oversight	29.76%
3. The combined burden of domestic and external work	27.38%
4. Collaborative and defined support roles	10.12%
5. Specialized needs and circumstances	1.79%

Source-sample survey, 2025.

The pre-dominant theme, identified in 32.14% of instances, is the Complete Substitution of Primary Parental Responsibilities. This suggests that around one-third of grandparents undertake the full, continuous responsibilities of a parent. Their responsibilities include comprehensive physical care, including bathing, feeding, and cleanliness, in addition to offering primary emotional support to compensate for the absent parent. The second pre-dominant theme, observed in 29.76% of instances, is Logistical Management and Academic Oversight. This indicates that a substantial segment of caring is centered exclusively on children's education, with grandparents serving as full-time drivers, academic overseers, and representatives at school meetings. The third pre-dominant topic is the Burden of Domestic and External Work, identified in 27.38% of instances. This indicates that childcare is not a standalone responsibility but rather interwoven with the grandparents' existing, burdensome obligations of household administration and, in certain instances, income-generating endeavors, leading to a significant deficiency in personal leisure time. A smaller section of caring is identified as more supportive or specialized. The subject of Collaborative and Defined Support Roles is present in 10.12% of cases. This result outlines a situation in which the grandmother is not the exclusive guardian but offers crucial support to a resident parent who is employed locally, frequently doing particular, time-sensitive responsibilities such as nighttime childcare or food preparation. The least prevalent yet most intensive theme is Specialized Needs and Circumstances, comprising 1.79% of cases. This discovery indicates a rigorous caregiving model centered on children with health complications or impairments, necessitating grandparents to oversee medicines, participate in numerous medical consultations, and offer specialized developmental assistance.

### 3.5 Mental stress among caregiving grandparents

**Figure 6:**
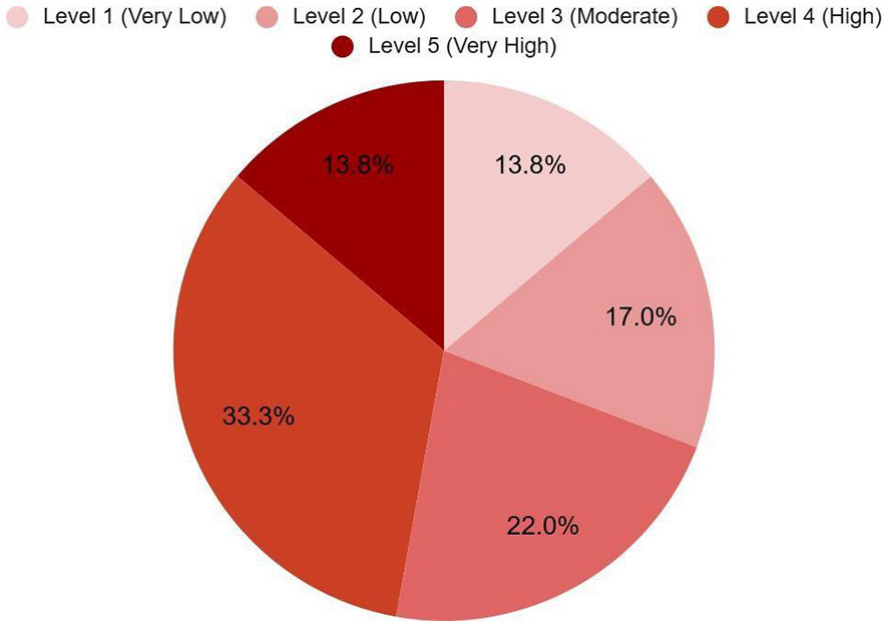
Self-reported stress levels (*N* =163). Source-sample survey, 2025.

Self-reported stress levels reveal that high stress is the predominant experience for caretaking grandparents in transnational households (Figure 6). The single most frequently reported category is “High” stress, accounting for one-third (33.3%) of the population. Cumulatively, a substantial portion (47%) of all grandparents report experiencing either “High” or “Very High” levels of stress. This figure overshadows the 31% who report “Low” or “Very Low” stress, suggesting that elevated psychological strain is a normative, rather than exceptional, feature of this demanding caregiving role. Based on the responses, the causes of high and very high stress among caretakers are categorized into five distinct areas based on the responses provided by 82 respondents who reported high and very high stress levels (Table 3).

**Table 3:** Causes for high stress levels (level 4 and 5) and their frequencies (*N* = 82).

Cause for high stress (level 4 and level 5)	Percentage
Financial and economic pressure *Stress resulting from profound poverty, debt, inconsistent financial support from the migrant child, the high cost of living, and the anxiety related to providing for the family's basic needs. This is often the root cause of the entire migratory arrangement*.	18.30%
Security and worry about the migrant child *High anxiety focused specifically on the migrant child, usually a daughter, due to concerns over her safety, the hardships she endures while working abroad, the physical distance, and the danger inherent in her job or location*.	17.10%
Managing difficult grandchild behavior and care load *Stress caused directly by the challenges of caregiving, including managing naughty, playful, or stubborn grandchildren; dealing with the logistical burdens, such as daily school runs; and the sheer volume of work (doing all household and child tasks oneself)*.	26.80%
Caregiver's own health and physical strain *Stress directly linked to the caregiver's own physical limitations, age, or health issues (such as diabetes, pressure, heart disease, or general illness) while simultaneously dealing with the demanding physical requirements of looking after active children*.	17.10%
Family conflict and instability (spouse/parent issues) *Stress arising from internal family problems, such as the drinking or behavior of the remaining son/spouse (the grandchild's parent); separation or absence of the daughter's husband; or conflict arising from the family's social circumstances, such as divorce or separation*.	17.10%
Educational inability and anxiety *Stress caused by the grandparents' perceived inability or lack of knowledge to assist the grandchildren with complex modern academic tasks, leading to anxiety about the child's future educational success*.	3.70%

Source-sample survey, 2025.

### 3.6 Duration of caregiving responsibilities among grandparents

**Figure 7:**
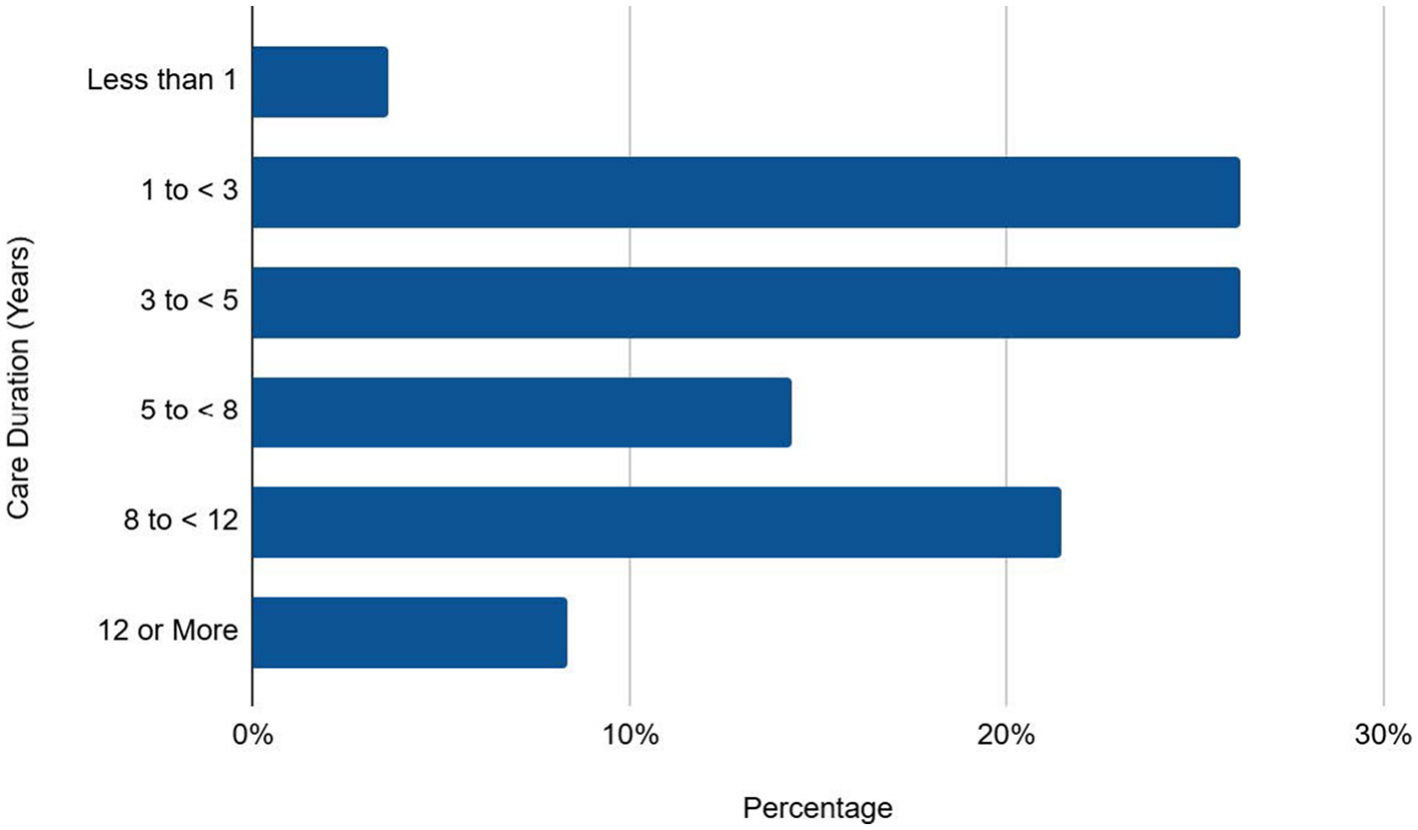
Duration of grandparental care (*N* = 163). Source-sample survey.

A significant majority of care arrangements fall within the 1 to 5 year range (Figure 7). The most common durations are 1 to less than 3 years and 3 to less than 5 years, each accounting for 26% of all cases. Combined, this means over half (52%) of grandparents provide care for their grandchildren for a period between1 and 5 years. Very short-term care, defined as less than 1 year, is relatively uncommon, representing only 4% of the total observed durations. Despite the prevalence of these medium-term arrangements, a notable proportion of grandparents provide extended care. Nearly one-third (29%) of care durations last 8 years or longer. This includes 21% of cases where grandparents provide care for 8 to less than 12 years or less, and 8% where they provide care for 12 years or more, either as primary or secondary caregivers in transnational households.

### 3.7 Grandparents' perceptions of caregiving

Participants were requested to articulate their sentiments regarding their children relocating overseas and entrusting their care to others, with the comments categorized into five principal topics. The prevailing mood among these caregivers is one of Positive Emotions (Happiness, Trust, and Satisfaction), articulated by roughly 30% of respondents. A sense of personal fulfillment is obtained from their role. Grandparents often highlight the profound confidence their children bestow onto them, perceiving their caregiving as “optimal care and affection.” This cultivates a sense of “humble pride” and genuine satisfaction in having their grandchildren in their lives.

Approximately 24% of grandparents are primarily motivated by a profound sense of Duty, Responsibility, and Sacrifice. These caregivers perceive their work as an essential duty undertaken for the future prosperity and welfare of the younger generation. They unequivocally accept the “significant responsibility” assigned to them, perceiving their endeavors as a vital contribution to their migrant children's quest for economic stability and their grandchildren's enhanced prospects.

A primary factor influencing these care arrangements is Economic Necessity and Absence of Alternatives, reported by nearly 19% of caregivers. This theme emphasizes that migration and the resulting caring arrangement frequently stem from impoverishment and a significant lack of alternatives. Grandparents recognize that migration transpires “due to poverty” or because parents had “no means to generate” revenue. The caring position is perceived as indispensable until the migrant parent attains “economic stability” overseas, since “no alternative support” exists.

Notwithstanding the favorable elements and profound sense of obligation, a significant percentage of grandparents encounter the challenges, burdens, and limitations of their role, comprising 16% of replies. This theme addresses the physical and psychological burdens of caring for small children in advanced age. Caregivers often express grievances regarding “additional responsibilities and physical discomfort” and bemoan the significant impact on their personal autonomy. Their previously solitary existence has now become hectic, and many articulate a need for respite, aspiring to “live a liberated life soon.”

A significant portion of grandparents conveys Concern, Sorrow, and Disapproval, accounting for little more than 11% of the perspectives. This illustrates the psychological toll of transnational familial separation. These caregivers express profound sorrow regarding the arrangement, frequently perceiving it as an injustice to the child, who is robbed of direct parental affection. This mental turmoil is exacerbated by concerns for the migrant child's safety in potentially hazardous working conditions overseas.

### 3.8 Support available to grandparents

**Figure 8:**
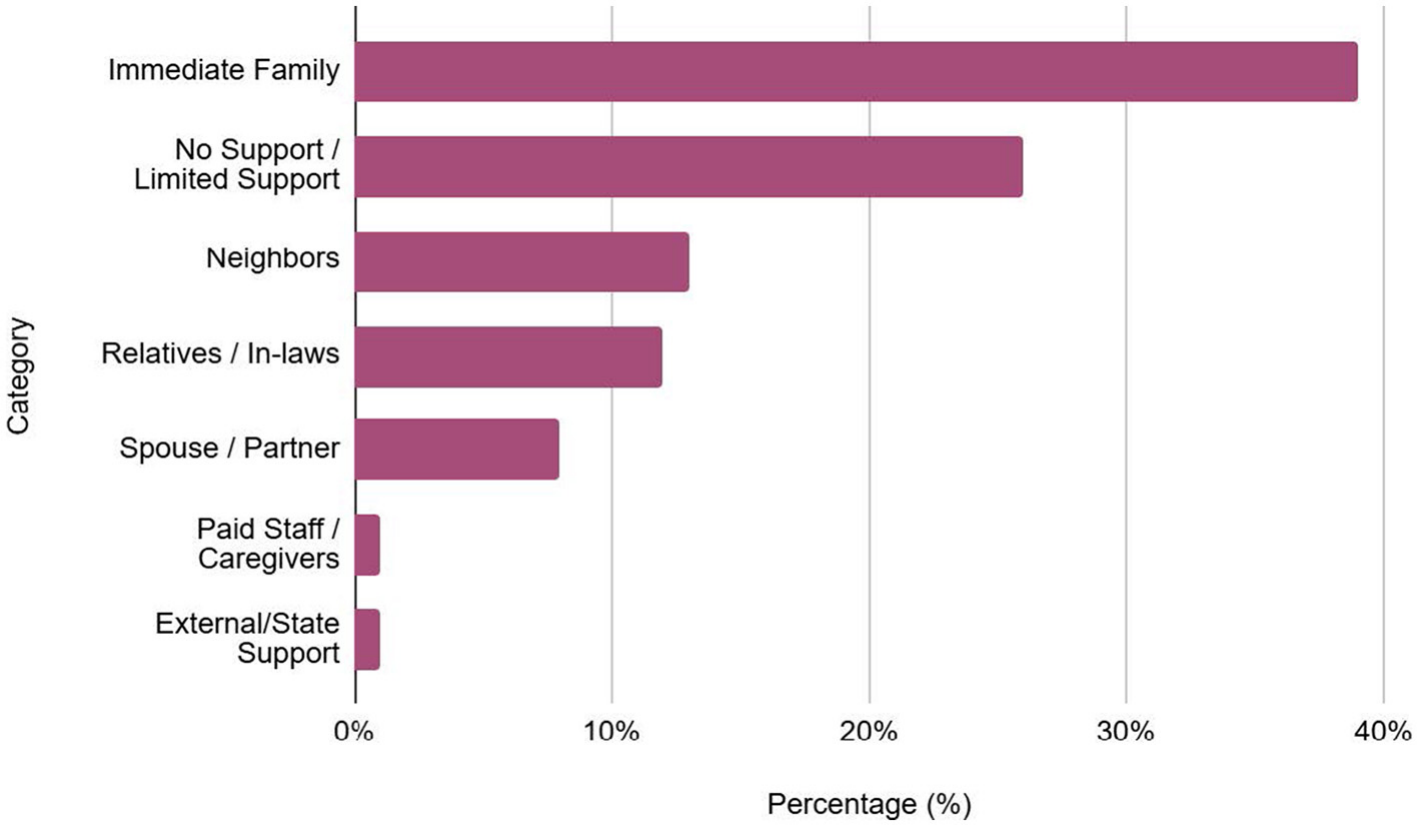
Sources of support available to caregiving grandparents (*N* = 163). Source-sample survey.

According to the responses, the most substantial source of support comes from the Immediate Family, accounting for a considerable 39% of cases (Figure 8). This indicates that other members within the grandparent's household or very close kin are involved in sharing the caregiving responsibilities. However, a concerning finding is that over a quarter of grandparents (26%) report receiving No Support or Limited Support. This suggests a substantial segment of grandparent caregivers is shouldering the immense burden largely on their own, facing potential isolation and increased strain in their caretaking role.

Beyond the immediate household, informal community networks also play a supportive, albeit secondary, role. Neighbors assist in 13% of situations, closely followed by Relatives/In-laws who contribute support in 12% of cases. These figures shed light on the importance of local community ties and extended family in buffering the caregiving load, especially in the absence of more formal structures.

Support from a Spouse/Partner is reported by a smaller segment, at 8%. This indicates a prevalence of single-grandparent caregivers, widowed individuals, or situations where the spouse themselves is elderly or unwell, limiting their capacity to assist.

Notably, formal or paid support systems are almost absent from these networks. Paid Staff/Caregivers and External/State Support each contribute a mere 1% to the overall support structure.

This highlights the critical lack of institutional safety nets, financial resources, and dedicated programs available to these grandparent caregivers, forcing them to rely almost entirely on their personal networks and resilience.

#### 3.8.1 Support from grandchildren

**Figure 9:**
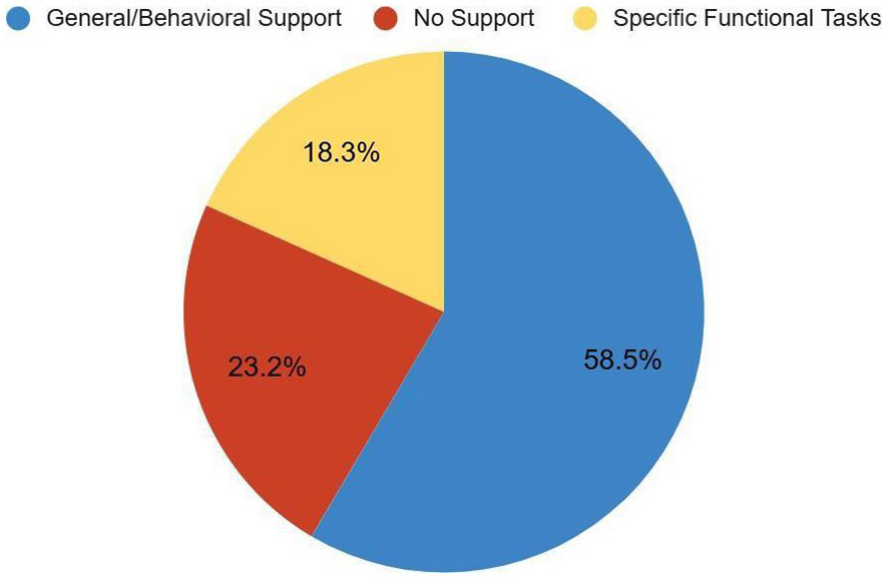
Support from grandchildren (*N* = 163). Source-sample survey, 2025.

Grandparents were asked about the kind of support they receive from grandchildren, since their input can have a varying impact on the volume and the nature of work grandparents have to engage in the absence of their parents (Figure 9). The most significant contribution from grandchildren, cited by a substantial majority of grandparents at 58.5%, falls under General/Behavioral Support. This category emphasizes the relief grandparents derive from their grandchildren's independence, obedience, and ability to manage their own affairs. Caregivers specifically value children who “manage their own tasks,” “are obedient,” and “cause no trouble,” as this may significantly reduce the daily demands and management burden on the often-elderly caregivers.

Conversely, a considerable portion of caregivers (23.2%) report receiving No Support from their grandchildren. This lack of contribution is frequently explained by the grandchild's age (“too young/small”) or their pre-occupation with personal activities like academic studies or entertainment.

Direct, hands-on assistance with household duties is the least common form of support. Only a small minority, 18.3%, of caregivers receive help with Specific Functional Tasks. These involve tangible contributions to basic chores such as cooking, cleaning, sweeping, laundry, or running small local errands. This low percentage underscores that physical help with daily chores from grandchildren is infrequent, further emphasizing the primary reliance on the grandparents themselves for these duties. The primary form of “help” is indirect, stemming largely from the grandchildren's self-sufficiency and good behavior, rather than direct assistance with household chores.

#### 3.8.2 Remittances and family contact

Regarding remittances and financial support, the vast majority of migrant children, nearly 80%, consistently send money every month to their families, ensuring a regular and reliable income flow. Only a small fraction, 17%, provides periodic or conditional support, with an almost negligible 4% offering irregular or non-existent financial contributions. This strong pattern of regular remittances underscores the migrant children's commitment to the economic stability of their families back home.

Furthermore, this financial commitment extends to the grandparents' health needs. A higher proportion of migrant children, 84%, regularly provide financial support directed at the health of their parents, who are acting as caregivers. This specific focus on health suggests a deep concern for the wellbeing of the often-elderly grandparent caregivers. Intermittent health support comes from 10% of remitters, and a minor 6% provide no or insufficient funds for health-related expenses, indicating that the health of the primary caregivers is a significant priority for the migrant children.

Migrant children maintain a high level of connection with their households that extends beyond financial support. A substantial majority report consistent contact, either with high frequency (Daily/Near Daily at 45.50%) or at least weekly (51.30%). This demonstrates a consistent effort to stay in touch and engaged with their families despite geographical distance. Only a very small percentage (3.30%) shows infrequent, irregular, or poor communication. This sustained connection likely plays an important role in mitigating the emotional challenges associated with transnational separation for both the grandparents and grandchildren.

### 3.9 Perceived health impacts of caregiving

**Figure 10:**
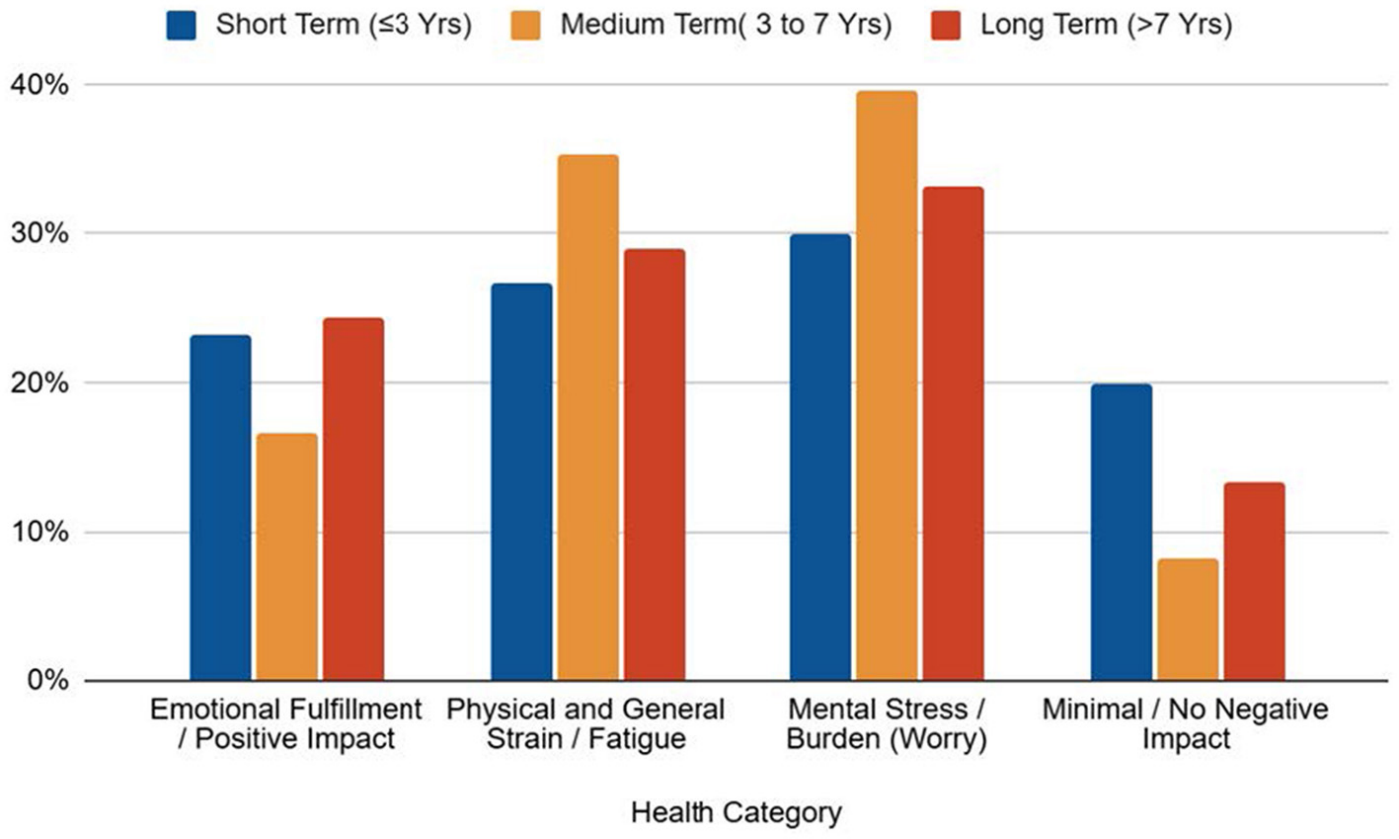
Perceived health impacts of caregiving (*N* = 163). Source-sample survey, 2025.

The influence of grandparental caregiving on health significantly differs depending on the period of care provided (Figure 10). Emotional Fulfillment/Positive Impact initiates at a robust 23% in the Short Term ( ≤ 3 years), declines significantly to 17% in the Medium Term (>3 to 7 years), and subsequently rebounds to 24% in the Long Term (>7 years). Conversely, adverse effects have a distinct increase with time. Physical and General Strain/Fatigue is first reported by 27%, increases to 35% in the Medium Term, and subsequently diminishes to 29% in the Long Term, indicating that the Medium Term represents a peak in physical challenge. Mental stress or burden (worry) constitutes the most persistent obstacle, commencing at 30%, escalating dramatically to 40% in the medium term, and sustaining a high level of 33% in long-term care. Consequently, the percentage of grandparents indicating Minimal or No Negative Impact significantly decreases from 20% in the Short Term to merely 8% in the Medium Term, remaining low at 13% in the Long Term. This suggests that although the initial phase may exhibit the most favorable sentiment and least strain, prolonged caregiving is pre-dominantly marked by heightened physical fatigue and enduring mental burden.

### 3.10 Daily life limitations due to caregiving

**Figure 11:**
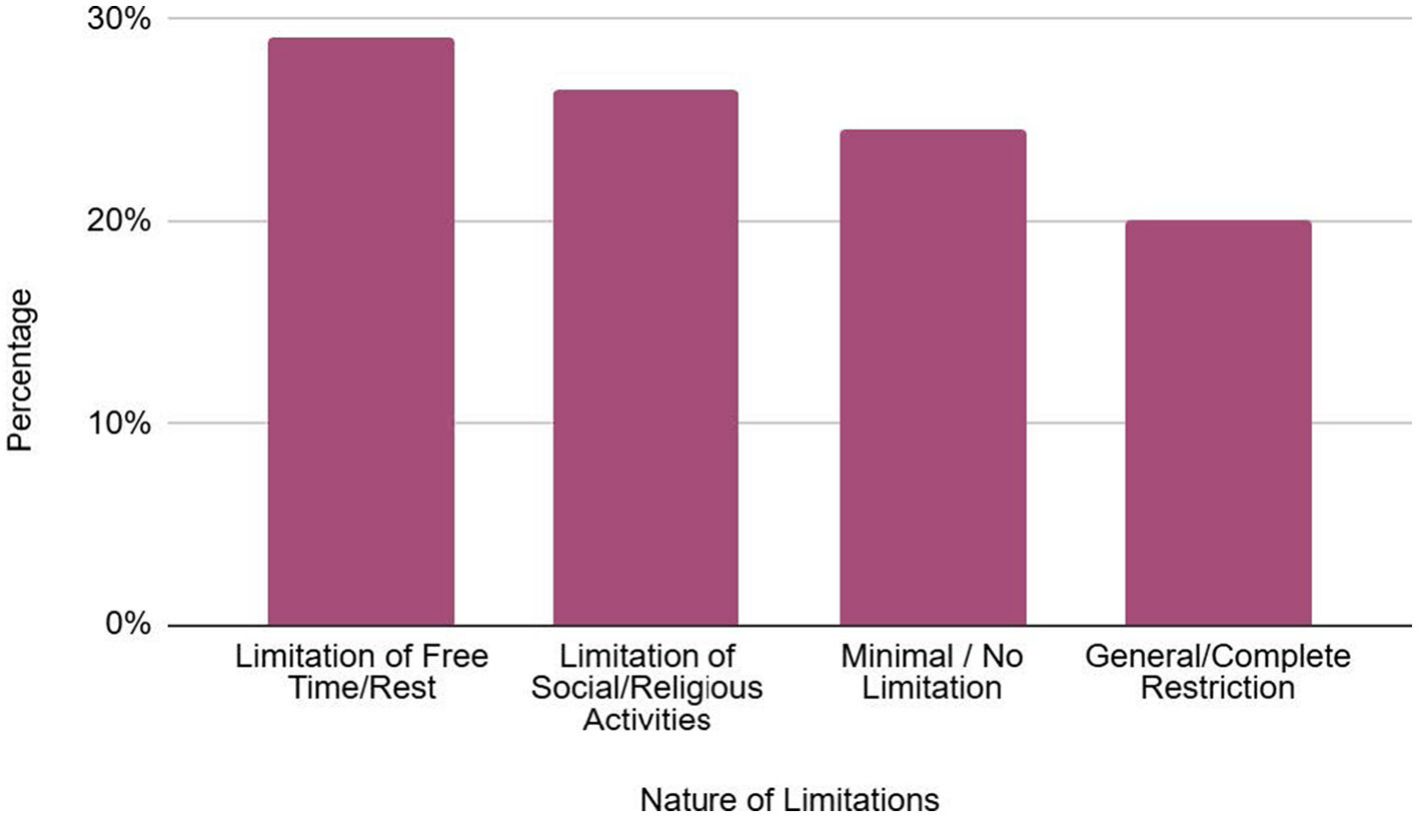
Limitations on daily life due to caregiving (*N* = 163). Source-sample survey.

The most frequently reported limitation, affecting around 33% of caregivers, is the Limitation of Free Time/Rest (Figure 11). The demanding, round-the-clock nature of constant caregiving, complicated by the caregivers' advanced age, likely eliminates opportunities for personal time, relaxation, or leisure activities that would typically be associated with retirement.

A substantial 27% of caregivers experience a Limitation of Social/Religious Activities. This means their caregiving duties restrict their ability to participate in community and spiritual life. They are often prevented from leaving home to attend religious events (such as Poya observance), engage with societies or clubs, or maintain social connections by visiting relatives and friends. Twenty five percent of caregivers report Minimal/No Limitation to their personal lives. These individuals indicate that they have either found adequate support systems, successfully integrated the children into their existing routines, or maintained a strong sense of personal independence, suggesting varied coping mechanisms or circumstances.

One-fifth of respondents convey a pervasive sense of General/Complete Restriction. These grandparents feel their lives and all prior activities, particularly their retirement plans, have been entirely sacrificed or dedicated to the needs of their grandchildren, leading to a perception of a total loss of personal freedom. The caregiving role imposes significant constraints on grandparents' personal lives, stripping them of free time, rest, and social/religious participation. While a quarter manage to avoid major limitations, a substantial segment feels their personal freedom has been entirely consumed by their caregiving duties.

### 3.11 Satisfaction in caregiving

**Figure 12:**
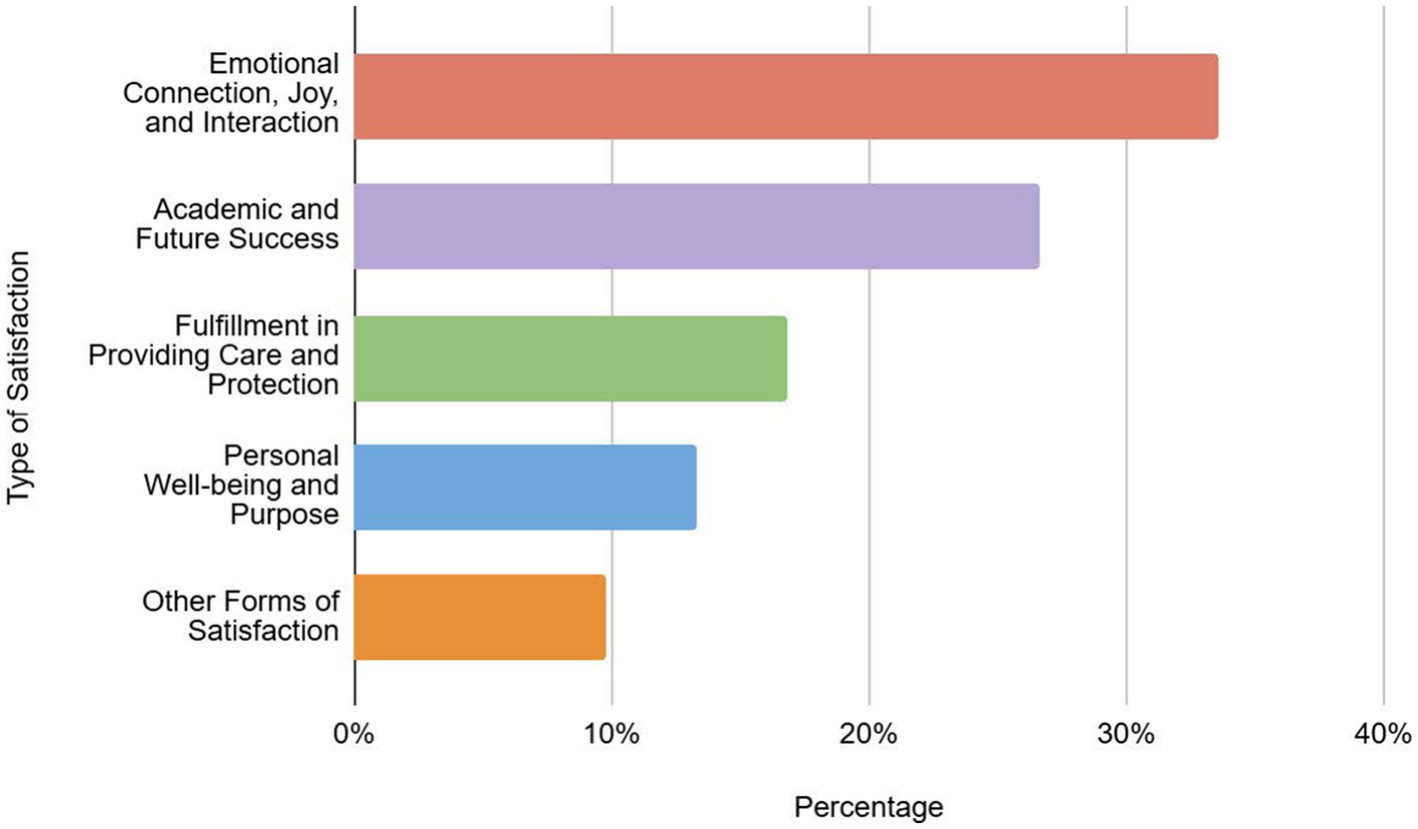
Sources of caregiving satisfaction (*N* = 163). Source-sample survey, 2025.

Grandparents were asked about the most rewarding part of the caretaking role, and the responses were divided into five categories that may have overlapping elements, given the open-ended nature of the question (Figure 12). The most significant source of fulfillment, reported by 34% of caregivers, is Emotional Connection, Joy, and Interaction. This highlights the happiness, mutual affection, and immediate emotional bonds derived from spending time and interacting directly with their grandchildren, emphasizing the joy found in their presence and the emotional connection.

Twenty seven percent of grandparents find satisfaction in their grandchildren's Academic and Future Success. This indicates pride and fulfillment derived from actively supporting the children's education and witnessing their academic achievements, seeing it as establishing a solid foundation for their long-term stability.

Another notable source of satisfaction for 17% of caregivers is the Fulfillment in Providing Care and Protection. This theme emphasizes the rewarding feeling of successfully fulfilling the critical role of primary caregiver, ensuring the children's safety, physical wellbeing, shelter, and guidance, and being a reliable, essential presence in their lives.

Furthermore, 13% of grandparents report positive impacts on their Personal Well-being and Purpose. The presence of their grandchildren enhances their own quality of life by alleviating loneliness, providing a renewed sense of purpose, and serving as a welcome distraction from personal illnesses or sadness.

Ten percent find satisfaction in Other Forms of Satisfaction, which includes specific, less frequently mentioned activities such as engaging in religious practices with the children, preparing their favorite foods, or observing unique character traits in their grandchildren.

### 3.12 Grandparents' attitudes toward adult children's migration

**Figure 13:**
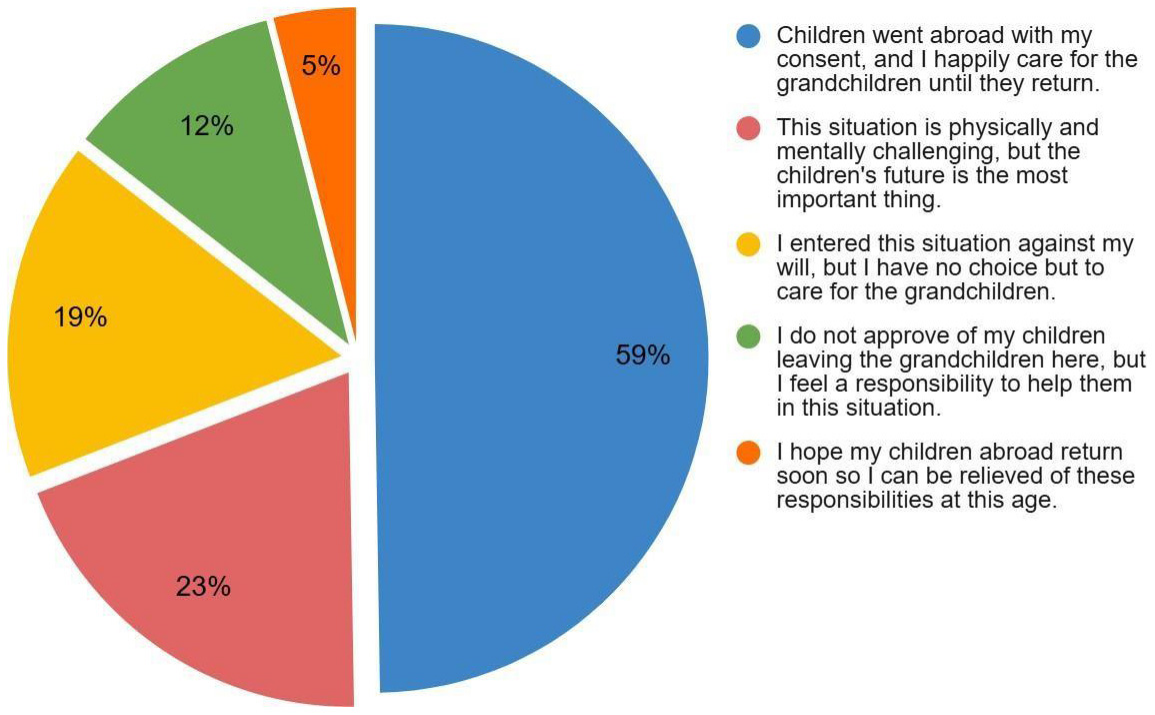
Grandparents' attitudes toward adult children's migration (*N* = 163). Source-sample survey, 2025.

Grandparents were asked to select the statement(s) that best described how they relate to the current care arrangements. It should be noted that the five attitude statements are not logically mutually exclusive. In particular, a respondent endorsing Statement 3, that they entered the arrangement against their will, would plausibly also endorse Statements 4 and 5, which express disapproval and a desire for relief, respectively. A significant majority of 59% of grandparents explicitly endorse their children's migration, showing a cheerful willingness to care for their grandchildren until the parents' return (Figure 13), indicating a foundational acceptance of the temporary caregiving arrangement.

However, even among those accepting the role, many acknowledge the difficulties. Twenty three percent of grandparents describe the situation as physically and mentally challenging, yet firmly prioritize their grandchildren's future above their personal comfort. This underscores a deep sense of responsibility and sacrifice for the next generation's wellbeing.

A notable portion of grandparents express reservations or a lack of personal agency in this arrangement. Specifically, 19% report entering the situation “against their will,” feeling they have no other choice but to care for their grandchildren. Complementing this, 12% state their disapproval of their children leaving the grandchildren, while still feeling an undeniable responsibility to help in the given circumstances. Five percent openly voice a desire for their children to return soon, so they can be relieved of these responsibilities.

It seems that while a majority of grandparents willingly embrace this caregiving role, often with an eye on the grandchildren's future, a significant number also grapple with feelings of obligation, physical strain, and a longing for relief. It should be noted that since statements are not mutually exclusive, Grandparents can relate to more than one description at the same time.

## 4 Discussion

This study suggests that Sri Lankan grandparents acting as caregivers in migrant households encounter a multifaceted blend of pride and stress. Many responders expressed positive sentiments regarding their role. Approximately 30% reported experiencing sentiments of contentment, trust, and fulfillment, frequently highlighting the pride associated with caring for grandchildren and the trust bestowed upon them by their adult children residing overseas. Nearly one-fourth characterized caring as an obligation or sacrifice made for the family's future. This aligns with studies from other Asian contexts, where grandparental caregiving is perceived as a culturally anticipated contribution that might provide renewed meaning (43). Conversely, the majority of caregivers in our sample indicated heightened stress and difficulty. Self-reported stress levels were primarily elevated. Approximately one-third assessed their stress levels as “High,” while over fifty percent classified their stress as either “High” or “Very High.” This widespread psychological stress indicates that extensive caregiving in later age is often challenging. Previous studies also report considerable anxiety and depressed symptoms in grandparent caregivers (3, 48). Consequently, our results align with existing literature, indicating that skipped-generation caregiving encompasses both emotional benefits and persistent stress.

The mental distress experienced by grandparents was ascribed to many variables. Financial instability was a primary issue: numerous caregivers contend with debt, elevated living expenses, and irregular remittances from overseas. Concerns regarding the safety of migrant children were almost equally prevalent. Grandparents often expressed concern regarding the long-distance care of their children employed in perilous environments. Challenging conduct or requirements of grandkids also played a role; over 26% of extremely stressed caregivers attributed their difficulties to the demands of managing energetic grandchildren and all household responsibilities independently. Significantly, the deteriorating health of grandparents was a stressor for 17% of individuals experiencing high levels of stress. The findings correspond with recent research indicating that financial stress, anxiety regarding distant relatives, and personal health challenges intensify grandparents' anxiety and sense of obligation (45, 51). Specifically, ongoing concern for family welfare is recognized to detrimentally affect mental and physical health, as corroborated by our qualitative data: numerous grandparents expressly reported experiencing anxiety and tension in their everyday lives (see also 3).

A key discovery pertains to the characteristics and intensity of caregiving responsibilities assumed by grandparents. Approximately one-third of participants indicated complete replacement of parental roles, undertaking the entire responsibility for children's physical care, emotional support, supervision, and discipline. This verifies that numerous Sri Lankan migrant homes function as skipped-generation households, wherein grandparents serve as de facto parents for extended durations (33). Our observations further reflect a distinct matrilateral bias in these caregiving arrangements, a phenomenon heavily magnified by the realities of transnational migration. Consistent with prior sociological observations (37, 38), the pre-dominance of daughters among migrants is consistent with the matrilateral bias documented in the broader literature (37, 38). This highlights the reliance on strong intergenerational mother-daughter solidarity to secure trustworthy care while the mother works abroad (5, 28). In contrast to temporary childcare assistance, these arrangements frequently persist for several years, mirroring the prolonged nature of abroad employment and the systemic reliance on migrant labor in Sri Lanka.

Furthermore, nearly one-third of grandparents characterized caregiving as focused on logistical coordination and academic supervision, encompassing school transportation, homework monitoring, and engagement with educational institutions. In Sri Lanka, where scholastic achievement is regarded as the principal pathway to social advancement, these obligations bear a significant emotional burden. Numerous grandparents conveyed apprehension regarding their restricted ability to assist grandchildren academically, especially concerning the contemporary curriculum. Comparable apprehensions have been noted among senior caretakers in other contexts, with educational supervision recognized as a main source of stress (52).

In addition to stress, physical health issues exacerbate the difficulties faced by grandparents. The survey revealed a prevalent occurrence of chronic illness among caregivers. Approximately 20% of both men and women reported hypertension, whereas over 25% reported diabetes. Significantly, grandmothers exhibited particularly elevated diabetes prevalence, with 32.4% of female caregivers affected. Arthritis and back discomfort were prevalent, affecting approximately 20–24% of individuals. Ultimately, this intersection of cultural expectation and biological pre-disposition dictates that the exhausting physical and emotional tolls of international migration's care deficits are disproportionately shouldered by aging women on the maternal side of the family (43). In reality, these diseases result in mobility restrictions, discomfort, and exhaustion that render the care of grandkids difficult. Grandparents frequently voiced apprehension regarding their capacity to maintain the demands of childcare as they grow older. Previous research has indicated that the physical requirements of raising, engaging with, and overseeing little grandkids can be straining (50). Our research indicates that Sri Lankan older adults frequently contend with various health concerns while fulfilling their caregiving responsibilities. The survey revealed significant comorbidity, with numerous respondents indicating two or more chronic diseases, possibly exacerbating the challenges of caregiving. The cumulative health burdens elucidate why a considerable proportion of grandparents in our sample perceived their role as a source of physical and psychological strain. The NCD rates in this sample are not markedly inconsistent with national elderly population estimates. Without a matched comparison group, causality cannot be established. The relationship is likely bidirectional existing illness makes caregiving harder, while caregiving stress may worsen health.

The grandparents' perceptions of the caregiving role differed. A significant percentage (about 30%) characterized the event positively, expressing delight and pride. This aligns with studies in South and Southeast Asia indicating that older persons frequently perceive the care of grandchildren as an intergenerational obligation (36, 43). In our findings, this frequently originated from the conviction that they are delivering the “optimal care and affection” and facilitating their migrant children's financial prosperity. Likewise, over 25% of respondents directly referenced obligation or sacrifice for the future of their grandkids. These findings correspond with the “family maximizer” idea in the literature: grandparents in migrant families intentionally offer childcare to enhance the family's socioeconomic chances (35).

Nonetheless, a significant minority of respondents conveyed ambivalence or discontent. Approximately 16% highlighted the challenges and demands of caregiving. These caregivers expressed grievances regarding “additional responsibilities” and physical discomfort, with many mourning the erosion of personal autonomy, characterizing their previously tranquil life as “now transformed into busyness” due to young children. Additionally, 11% expressed concern and sorrow at the agreement. They perceived it as unjust that children were denied their parents' affection and expressed concerns for the migrants' safety overseas. These sentiments show the emotional toll of transnational separation identified in previous research. Gamburd (31) and Caldwell et al. (52) discovered that migration might destabilize family dynamics and provoke intergenerational discord; grandparents may experience social isolation or depression when parental relationships are stressed. Some grandparents in our data reported experiencing stress with the remaining parent (e.g., wives of the migrants) or ambiguity regarding the child's education, reflecting problems noted in Caldwell et al. (52). Consequently, although numerous grandparents embrace their job, a notable portion perceives it as a taxing extension of parental sacrifice, indicating that good emotions frequently coexist with anxiety and exhaustion.

The support structures for these caregivers were pre-dominantly informal and constrained. In our survey, the pre-dominant source of assistance was other family members residing in the household (39% of cases). Extended family, including neighbors and in-laws, offered assistance in approximately 25% of instances. Nevertheless, more than one-quarter of grandparents indicated that they received no assistance beyond their own efforts, underscoring a potential risk of isolation. Assistance from a spouse or partner was infrequent (8%), frequently due to the fact that numerous grandparents were widowed or their partners were likewise advanced in age or unwell. Financial or governmental support was nearly absent, with about 1% indicating any official aid. This signifies that Sri Lankan grandparent caregivers rely pre-dominantly on personal and communal networks. This dependence aligns with other research in underdeveloped nations, where, due to insufficient benefits, households rely on familial connections (43). The absence of institutional safety nets in Sri Lanka results in the most vulnerable and isolated grandparents shouldering significant burdens independently, hence exacerbating the aforementioned stress.

Prolonged overseas labor migration, especially of mothers engaged in domestic work abroad, appears to have rendered skipped-generation families prevalent. Cultural norms significantly endorse grandparent participation: previous research indicates that in Sri Lankan (and wider South Asian) society, it is anticipated that grandparents will care for young grandchildren in the absence of parents. This practical reliance aligns seamlessly with biosocial theories of preferential investment, which demonstrate that maternal grandmothers possessing absolute relationship certainty consistently provide the highest levels of childcare (39, 40). The disproportionate burden of this caregiving falls to maternal grandmothers, a pattern that our data broadly corroborates. The present findings thus extend the existing literature (42) by demonstrating that this matrilateral bias operates within the specific Sri Lankan migration context. As a result, numerous grandparents perceive a duty to assume this position, even when it burdens them. The sense of “no alternative” signifies persistent poverty. Numerous individuals observed that migrating was imperative due to the absence of other means of income, and that caregiving responsibilities were inescapable till remittances were stable. In contrast to affluent nations with established childcare systems, Sri Lanka provides minimal public support for childcare or eldercare; the study's conclusion of nearly non-existent official assistance highlights this disparity. Collectively, our findings affirm that Sri Lankan grandparents encounter numerous challenges similar to those identified in worldwide studies on transnational caring (financial pressure, health deterioration, role conflict), but within a context of significant familial obligation and limited welfare resources. We did not identify significant discrepancies with previous research; instead, the results primarily serve as extensions: they bolster the literature on the challenges of skipped-generation caregiving (3, 46) while also demonstrating the culturally ingrained obligation and pride noted by certain scholars (43). The experiences of Sri Lankan grandparent caregivers illustrate the convergence of gender norms, economic need, social expectations, and profound matrilateral reliance in defining the roles and wellbeing of elders within migrant households.

## 5 Recommendations

This study's findings indicate that older grandparents in Sri Lankan migrant homes assume a pivotal caregiving role, frequently at the expense of their physical, psychological, and social wellbeing. Addressing the health and wellbeing of caregivers necessitates a multifaceted strategy that integrates healthcare access, psychosocial assistance, economic stability, and social acknowledgment.

Enhancing physical health outcomes for caregiving grandparents needs to be a goal. The significant incidence of chronic non-communicable diseases and age-related health constraints among caregivers underscores the necessity for proactive, age-sensitive healthcare provision. Enhancing primary healthcare services to incorporate regular geriatric evaluations, home-based monitoring, and medication management assistance can alleviate physical burden and avert health decline. Community health workers and public health midwives should have a more significant role in identifying caregiving grandparents and facilitating continuity of care. Regional research demonstrates that community-based geriatric health services effectively enhance health outcomes for older persons involved in unpaid caring (54, 55).

The provision of mental health and psychosocial support for caregiving grandparents is equally crucial. The research reveals elevated stress levels linked to caregiving duties, anxiety for migrating children, and ambiguity about the future. Incorporating fundamental mental health assessments and counseling within primary healthcare facilities can facilitate the identification of caregivers undergoing psychological distress. Furthermore, the formation of peer support groups within the community would enable grandparents to exchange experiences, mitigate isolation, and cultivate coping methods. Data from migration-impacted homes in South Asia indicates that psychosocial interventions can markedly enhance the emotional wellbeing of older caregivers (3).

The help offered to caregiving grandparents must also include their practical needs. Numerous grandparents engage in physically strenuous childcare activities with informal support or independently. Implementing community-based respite care initiatives, including part-time childcare assistance via trained volunteers or local childcare facilities, can alleviate the intensity of everyday caregiving responsibilities. Even minimal alleviation can beneficially influence physical and emotional health. Global research on caring highlights that respite support is an essential preventive element against caregiver burnout (56). Expatriates with families remaining in their home countries should be motivated to offer financial support, since they will also directly benefit from these programs.

Economic strain exacerbates caregiver wellbeing, underscoring the necessity for financial and material support systems. While remittances augment household income, they frequently fail to sufficiently address healthcare expenditures and everyday caregiving expenses. Developing income security via improved pensions, healthcare subsidies, or focused financial support for older caretakers would mitigate economic strain. Regional aging frameworks emphasize that income security and access to inexpensive healthcare are essential for sustaining wellness in later life (54, 57).

Assistance should additionally encompass the enhancement of familial and international relationships. Facilitating consistent communication between migrant parents and caregiving grandparents can promote a more equitable distribution of emotional responsibility and decision-making. Promoting collaborative caregiving choices and emotional engagement of migrant parents may alleviate stress and diminish feelings of isolation among grandparents. Studies on transnational families indicate that regular communication alleviates mental distress and enhances caregiving relationships.

Enhancing caregiver welfare necessitates more societal awareness and institutional validation of the caregiving roles of grandparents. Identifying senior caregivers within social welfare and healthcare systems might enhance service targeting and diminish their invisibility. Data gathering methods must include the caring status to guarantee that support programs effectively target individuals most in need. Cooperative involvement among healthcare professionals, social agencies, and community organizations is crucial for providing integrated support that addresses the intricate health and wellbeing requirements of caregiving grandparents in Sri Lanka.

## 6 Conclusion

This study aimed to investigate the experiences of older grandparents caring for left-behind children under 18 in migrant households, contextualizing their caregiving within the larger framework of labor migration and population aging in Sri Lanka. The findings suggest that grandparental caregiving is not a peripheral or transient family arrangement but a fundamentally integrated aspect of Sri Lanka's migration economy that facilitates the emigration of the working-age population. By performing primary or secondary caring duties, grandparents facilitate the participation of working-age people in international labor markets, hence sustaining remittance flows that greatly enhance household livelihoods and national economic stability.

The caregiving duties assumed by grandparents constitute a substantial yet pre-dominantly unrecognized aspect of social reproduction. The research indicates that older caregivers participate in extensive, prolonged caregiving that encompasses complete parental replacement, educational oversight, domestic administration, and emotional assistance. This unpaid caregiving is crucial for sustaining family dynamics across borders, although it occurs in the context of deteriorating health, insufficient formal assistance, and escalating physical and psychological stress. These findings underscore the disparity between the economic advantages gained from labor mobility and the overlooked burdens shouldered by older generations.

The research underscores the necessity to reframe the concept of aging beyond the narrative of dependency. Grandparents in migrant families significantly enhance both familial welfare and overarching socioeconomic dynamics. Their caregiving labor facilitates the intergenerational transfer of care, fosters the human capital development of the younger generation, and indirectly supports national growth through remittance-driven migration. Characterizing older individuals only as dependents disregards their productive and reproductive roles within homes and society.

The results must be understood considering specific constraints. The research relies on cross-sectional data and non-probability sampling, hence constraining generalizability and precluding causal inference. The lack of a reference group of senior caregivers in non-migrant families limits the capacity to discern migration-specific impacts. Future research would be enhanced by longitudinal designs, comparative comparisons of migrant and non-migrant households, and the use of qualitative methods to further investigate the long-term health, emotional, and social ramifications of caregiving in aging societies.

This research aims to further the understanding of migration and aging in Sri Lanka by examining the lived experiences of caregiving grandparents. It underscores that population aging and labor mobility are intricately linked processes, facilitated by family-oriented care arrangements. Recognizing and appreciating the caregiving contributions of grandparents is crucial for comprehending the actual societal costs and sustainability of economic migration in developing countries facing population aging, such as Sri Lanka.

## Data Availability

The raw data supporting the conclusions of this article will be made available by the authors, without undue reservation.
